# Facial Emotion Recognition in Parkinson's Disease: An fMRI Investigation

**DOI:** 10.1371/journal.pone.0136110

**Published:** 2015-08-18

**Authors:** Albert Wabnegger, Rottraut Ille, Petra Schwingenschuh, Petra Katschnig-Winter, Mariella Kögl-Wallner, Karoline Wenzel, Anne Schienle

**Affiliations:** 1 Clinical Psychology, University of Graz, BioTechMedGraz, Graz, Austria; 2 Department of Neurology, Medical University of Graz, Graz, Austria; Tilburg University, NETHERLANDS

## Abstract

**Background:**

Findings of behavioral studies on facial emotion recognition in Parkinson’s disease (PD) are very heterogeneous. Therefore, the present investigation additionally used functional magnetic resonance imaging (fMRI) in order to compare brain activation during emotion perception between PD patients and healthy controls.

**Methods and Findings:**

We included 17 nonmedicated, nondemented PD patients suffering from mild to moderate symptoms and 22 healthy controls. The participants were shown pictures of facial expressions depicting disgust, fear, sadness, and anger and they answered scales for the assessment of affective traits. The patients did not report lowered intensities for the displayed target emotions, and showed a comparable rating accuracy as the control participants. The questionnaire scores did not differ between patients and controls. The fMRI data showed similar activation in both groups except for a generally stronger recruitment of somatosensory regions in the patients.

**Conclusions:**

Since somatosensory cortices are involved in the simulation of an observed emotion, which constitutes an important mechanism for emotion recognition, future studies should focus on activation changes within this region during the course of disease.

## Introduction

Idiopathic Parkinson’s disease (PD) is associated with progressive degeneration of nigrostriatal dopaminergic pathways. Clinically, this leads to the typical motor symptoms. In addition, difficulties in affective processing have been reported for this patient group. Facial emotion recognition is one of the most widely studied affective capabilities within this context. Surprisingly, studies focusing on behavioral indicators of identification and discrimination accuracy of facial affect found very heterogenous results for PD patients (for a review see [1]). Some research groups observed a marked general impairment [2–4], whereas others reported problems for specific emotions [5–7], or no deficit at all [8–10].

The differences in responding are possibly a result of moderating factors such as stage of disease, medication status, and comorbid depression. Often patients were tested who greatly varied with regard to symptom severity, which is correlated with visual and executive dysfunctions [9]. In the majority of studies patients receiving dopamine replacement therapy or dopamine agonists were studied, whereas experiments which included nonmedicated patients are still limited [2, 4, 6, 11]. Comorbid disorders such as depression have a confounding effect on the decoding of facial affect. Very frequently depressive symptoms were not sufficiently controlled (e.g., some authors did not exclude patients with mild depressive symptoms or sometimes depressive symptoms were not reported at all [1]).

Finally, although behavioral measures are valuable indicators of affective processing neurobiological measures might be more sensitive. The number of experiments which used brain imaging techniques such as functional magnetic resonance imaging (fMRI) as additional indicators of facial emotion processing is however very low. Tessitore et al. revealed that PD patients lacked amygdala activation when viewing facial expressions of fear 22 [12]. This effect was present in the hypodopaminergic state and could partially be restored by dopamine repletion. In a more recent fMRI study by Anders et al. (2012) asymptomatic Parkin mutation carriers were presented with affective facial expressions (happiness, fear, disgust, anger, sadness, and surprise) [13]. Relative to healthy controls, the mutation carriers showed a comparable recognition accuracy for ‘clear-cut’ affective expressions, but their recognition performance was decreased for ‘fuzzy’ emotional faces. In addition, the mutation carriers were characterized by increased brain activity in the right ventrolateral premotor cortex/ pars opercularis while exposed to positive facial expressions. To the best of our knowledge there are no other fMRI experiments on facial emotion recognition in PD.

Therefore, in the present fMRI investigation we studied patients, who had discontinued medication for 10 to 12 hours prior to the study. They showed no signs of depression and cognitive impairment. They were shown pictures with facial displays of basic emotions (disgust, fear, anger, sadness), and neutral facial expressions. The affective ratings for the images and the brain activation were compared between a clinical and a healthy control group. Moreover, we investigated possible group differences concerning affective traits (disgust proneness, trait anxiety, trait anger), which influence the processing of the mentioned specific emotion elicitors. The present study had an exploratory approach.

## Materials and Methods

### Subjects

Seventeen PD patients and 22 healthy controls participated in this study. All patients had been diagnosed with idiopathic PD by neurologists of the University Hospital in Graz (Austria). The clinical and the control groups did not differ in mean age, years of education, and did not show signs of cognitive impairment (Table 1). Cognitive performance had been assessed with the Test for Early Detection of Dementia (TEDD), a specific and sensitive screening instrument for mild dementia [14]. The TEDD comprises immediate and delayed recall, the clock drawing test, long term memory, temporal orientation, following commands, and verbal fluency. The scores range from 0 to 50 points. The cut-off score is 35 points indicating a tentative dementia diagnosis.

**Table 1:** Overview of descriptive data for patients with Parkinson’s disease (PD) and the control group (CG).

	PD M (SD)	CG M (SD)	*t value (df)*	*p* value
Sex (frequency)	8 f, 9 m	11 f, 11 m		
Age (years)	55.2 (9.4)	51.8 (9.8)	1.12 (37)	.272
Education (years)	13.1 (3.1)	13.8 (4.1)	-6.01 (37)	.424
TEDD	46.2 (1.9)	44.6 (3.19)	2.00 (35.2)	.053
Affective traits				
QADP	1.92 (0.44)	1.99 (0.53)	0.46 (37)	.650
BDI	9.35 (7.77)	6.55 (4.39)	1.33 (23.8)	.195
STAI	39.24 (11.7)	32.73 (9.02)	1.96 (37)	.057
STAXI	17.24 (4.14)	17.41 (4.17)	0.13 (37)	.898
Affective ratings: intensity				
Anxiety	6.44 (1.46)	5.44 (1.98)	1.71 (36)	.096
Disgust	6.16 (1.47)	5.63 (1.97)	0.90 (36)	.376
Sadness	7.43 (1.32)	6.33 (1.69)	2.15 (36)	.038
Anger	7.82 (0.87)	7.52 (0.96)	0.98 (36)	.331
Affective ratings: accuracy				
Anxiety	3.50 (1.74)	2.64 (1.86)	1.47 (33.7)	.152
Disgust	3.10 (1.22)	2.96 (2.38)	0.24 (32.9)	.814
Sadness	4.86 (1.56)	3.88 (1.82)	1.74 (36)	.091
Anger	5.34 (1.20)	5.60 (1.53)	-0.56 (36)	.580

f: female, m: male; TEDD: Test for Early Detection of Dementia; QADP: Questionnaire for the Assessment of Disgust Proneness; BDI: Beck Depression Inventory; STAI: State Trait Anxiety Inventory (trait scale); STAXI: State Trait Anger Inventory (trait scale).

The scores on the rating scale by Hoehn & Yahr [15] were either 2.0 (14 patients) or 2.5 (3 patients). Eleven PD patients had right body side onset of motor symptoms and 6 had left-side onset. The patients had a UPDRS [16] sum score of M = 36.1 (SD = 13.0) ranging between 17 and 49. This implies mild to moderate motor impairment. The symptom duration was on average M = 75.4 months (SD = 43.7).

With one exception all patients were treated with L-Dopa and/ or a dopamine agonist (pramipexole, ropinirole). Medication was discontinued for 10–12 h (overnight) prior to the fMRI session.

### Ethics

Written informed consent was obtained from each participant. The study was carried out in accordance with the Declaration of Helsinki and had been approved by the ethics committee of the Medical University of Graz.

### Questionnaires

All participants answered the following trait scales: *The Questionnaire for the Assessment of Disgust Proneness* (QADP) measures disgust propensity and describes 37 situations, which have to be judged on 5-point scales with regard to the experienced disgust (0 = ‘not disgusting’; 4 = ‘very disgusting’) [17]. The Cronbach’s alpha of the total scale is .90. The trait scale of the *State-Trait Anger Expression Inventory* (STAXI) assesses the tendency of a person to experience anger [18]. The Cronbach’s alpha is .87. The trait scale of the *State-Trait Anxiety Inventory* (STAI) measures the frequency of anxious feelings [19]. The questionnaire consists of 20 items which are answered on a 4-point scale (1 = ‘almost never’, 4 = ‘almost ever’). The Cronbach’s alpha of the scale is .88. *The Beck Depression Inventory* (BDI) consists of 21 items rated on 4-point scales [20]. A sum score of 18 or higher indicates clinical relevance, because this value is two standard deviations above the mean of the healthy reference sample. The Cronbach’s alpha of the scale is .89 [20]. All mentioned Cronbach’s alpha values refer to the reference samples of the corresponding test manuals.

### Stimuli and design

The participants were presented with a total of 50 pictures from the Karolinska Directed Emotional Faces set [21]. Due to higher statistical power compared to an event-related design we conducted a block design. Every block consisted of 10 pictures of the same emotional expression (disgust, sadness, anger, fear, or neutral). Each picture was presented for 3 seconds. This resulted in a total block length of 30 seconds. Each stimulus block was presented in a pseudo-randomized order for all participants and was repeated twice. It was ensured that none of the condition was followed by the same condition again. Further, each block was preceded by a fixation cross (5 seconds). In a gender discrimination task participants had to rate for each picture whether the shown person was male or female by pressing a button on a two-button device simultaneously to the presentation of the faces. This design is in line with previous studies examining emotional perception [22–24]. The rating accuracy was perfect in both groups. The total experiment lasted approximately 5 minutes. The short duration allowed the unmedicated patients to stay still during scanning; none of the patients had to be excluded from the sample due to movement artefacts.

Subsequent to the fMRI experiment, the participants gave affective ratings for the pictures. For each image they indicated the intensity of expressed disgust, fear, sadness, and anger (*Please indicate how intensely the depicted person experienced disgust/ fear/ anger/ sadness*: 1 = ‘very little’, 9 = ‘very intense’). Moreover, we calculated the classification accuracy, which was defined as the difference between the perceived intensity of a target emotion and the mean intensity of all non-target emotions for a specific facial expression (e.g., disgust classification accuracy for a disgust expression = disgust intensity minus mean intensity of non-target emotions [anger, fear, sadness]).

### fMRI: recording and analysis

Brain images were acquired using a 3 Tesla Siemens TrioTim (Siemens, Erlangen, Germany) with a 12-channel headcoil. For the functional runs a total of 164 volumes were acquired by using an echo-planar imaging protocol (35 descending slices; slice thickness: 3mm; TE = 30ms; TR = 2300ms; Voxel size: 3.0x3.0x3.0 mm; FoV: 192; flip angle: 90°; slice orientation -25° tilted from the AC-PC line). To account for saturation effects 3 slices from the beginning of the time series were discarded.

All analyses were conducted using SPM12 (Wellcome Department of Cognitive Neurology, London). For compensating field inhomogeneity we applied a fieldmap during the pre-subtracted phase and magnitude step in SPM12. Afterwards images were motion corrected via realignment, and to account for acquisition timing subsequent we set a slice timing step. Individuals’ t1 images were co-registered to the functional mean image and finally segmented into gray matter (GM) and white matter (WM). To create a study specific template and to increase inter-subject alignment a ‘Fast Diffeomorphic Registration Algorithm’ (DARTEL) was executed with GM and WM images. Resulting images were further normalized to MNI-space (3 mm isotropic voxel), and smoothed with an 8 mm isotropic Gaussian kernel. Data were high pass filtered (128 seconds) and temporal sphericity was controlled by an AR(1) process with consecutive pre-whitening of the data.

In the first level analysis we computed t-contrasts for different conditions (Fear > Neutral, Disgust > Neutral, Sadness > Neutral, Anger > Neutral). For a comparison of patients and control participants resulting images were afterwards submitted to a two sample t-test comparing voxel intensities. Furthermore, multiple regressions were conducted to correlate emotion intensity and accuracy ratings with activation in the selected ROIs. Based on previous findings on facial affect recognition we conducted a region of interest (ROI) approach for the following areas: amygdala, insula, orbitofrontal cortex (OFC), inferior frontal gyrus, basal ganglia, primary/ secondary somatosensory cortex (SI, SII), and inferior parietal cortex. The uncorrected height threshold for the analyses was set to *p* < 0.05. Voxel-peaks are reported when *p* corrected for family-wise error (FWE) < 0.05 (small volume correction). The ROI masks were created using the WFU Pickatlas (v2.4; Wake Forest University School of Medicine) and were taken from the Harvard-Oxford Cortical and Subcortical Structural Atlas (Center for Morphometric Analysis, MGH-East, Boston/MA, USA) and from the SPM Anatomy Toolbox [25].

Prior to the analysis of the functional MRI data, we had conducted a voxel-based morphometry (VBM) analysis in order to detect possible brain atrophy in the clinical group. The analysis was conducted with the VBM 8 toolbox. We used modulated images with a spatial smoothing of 10 mm. Further, we retained the default settings as described in the VBM manual. The uncorrected height threshold was set to *p* < .05 and the voxel-peak threshold to *p* < .05 (FWE-corrected). For the ROI analyses a small volume correction was applied. The conducted two-sample t-tests revealed no significant group differences neither for the chosen ROIs nor for the whole brain.

## Results

### Questionnaire scores and affective ratings

The two groups did not differ in their scores on the affective trait measures (disgust proneness, trait anxiety, trait anger, depressive tendencies; see Table 1). The scores were all in the normal range with the exception of controls showing lower trait anxiety compared with the norm sample of the STAI (*t*(144) = 3.77, *P* = .001).

The patients reported similar intensity ratings for the expressed target emotions anxiety, disgust and anger as controls, but indicated higher values for sadness (Table 1). The computed indices of rating accuracy for each emotion category (fear, anger, disgust, sadness) showed no group differences (all *p’s* > .09).

### fMRI

**Fig 1:**
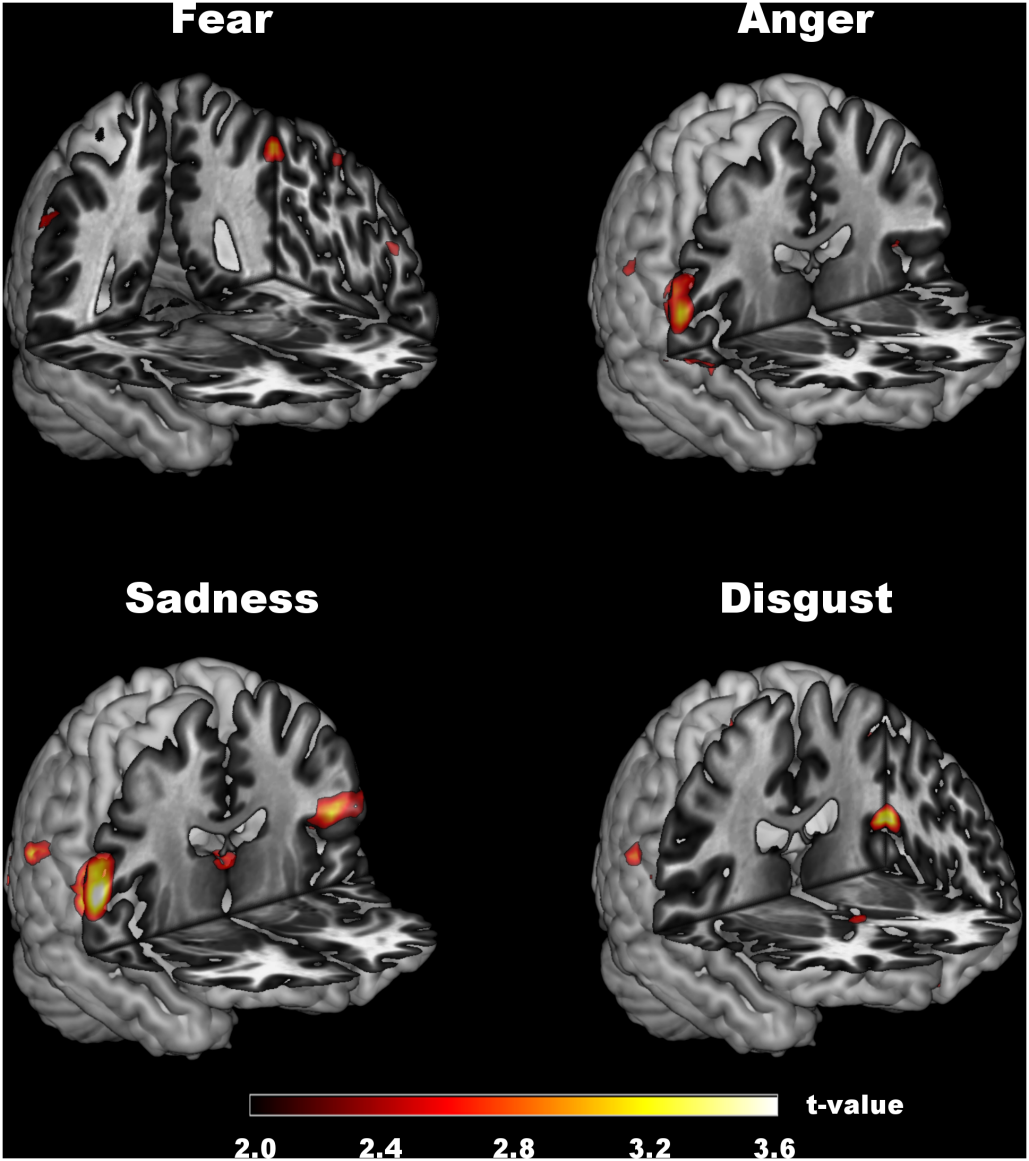
Regions with increased activation in patients compared to controls.

The group comparison showed that controls displayed greater activation in the putamen and inferior frontal gyrus when looking at sad faces (Table 2). PD patients were characterized by greater activation in parietal regions (SII, inferior parietal cortex) across all emotion conditions (anger, disgust, sadness, fear) than the control participants (Fig 1). An exploratory whole-brain analysis had revealed no significant group differences. Activation data separated for each group are displayed in S1 Table.

**Table 2:** Comparison of brain activation in the emotion conditions between patients with Parkinson’s disease (PD) and the control group (CG).

	H	X	Y	Z	*T*	*p*(FWE)	CS
**PD > CG: Anger > Neutral**							
Secondary somatosensory cortex (BA 22)	R	63	-12	15	3.22	0.037	36
**PD > CG: Disgust > Neutral**							
Inferior parietal lobule (BA 48)	R	54	-33	30	2.74	0.035	19
Secondary somatosensory cortex (BA 48)	L	-48	-21	21	3.38	0.032	59
**PD > CG: Fear > Neutral**							
Inferior parietal lobule (BA 40)	L	-51	-45	42	3.07	0.023	31
OFC (BA 45)	R	57	30	3	2.57	0.048	26
**PD > CG: Sadness > Neutral**							
Secondary somatosensory cortex (BA 22)	R	63	-12	15	3.74	0.011	34
Secondary somatosensory cortex (BA 48)	L	-51	-12	21	3.24	0.047	65
**CG > PD: Sadness > Neutral**							
Putamen (BA 48)	R	30	18	-9	3.49	0.047	38
Inferior frontal gyrus (BA 47)	R	30	21	-12	3.64	0.034	27

BA = Brodmann Area; H = hemisphere, MNI coordinates (x,y,z), *p*(FWE) = corrected for family-wise error, CS = cluster size (number of voxels).

In order to follow up on the enhanced parietal activation in the clinical group, we conducted correlation analyses in order to find out whether the intensity and accuracy ratings were associated with parietal recruitment. SII activation was positively correlated with fear intensity ratings (MNI coordinates: 51, -9, 21, *t* = 3.45, *p*(FWE) = .038, cluster size = 34) and accuracy ratings for fearful expressions (MNI coordinates: 54, -9, 18, *t* = 3,61, *p*(FWE) = .045, cluster size = 30). In addition, SII activation was positively correlated with disgust intensity ratings (MNI coordinates: -36, -27, 21, *t* = 3.15, *p*(FWE) = .032, cluster size = 18) and accuracy ratings for disgusted expressions (MNI coordinates: -36, -24, 15, *t* = 2.98, *p*(FWE) = .041, cluster size = 20). Anger intensity rating showed a positive association with inferior parietal activation (MNI coordinates: -48, -69, 33, *t* = 4.62, *p*(FWE) = .012; cluster size = 82).

## Discussion

This fMRI study investigated facial emotion recognition in a group of patients suffering from mild to moderate symptoms of idiopathic PD. The clinical group was free of depressive symptoms and suffered from no other comorbid disorders including cognitive impairment. Moreover, the medication had been discontinued for 10–12 hours prior to the investigation. Therefore, the affective ratings and associated brain activation during the picture processing were not influenced by these confounding variables.

The self-report of the patients indicated no deficit in affective processing, neither on the trait level nor on the state level. The patients and the control group had obtained comparable scores on the personality questionnaires assessing disgust proneness, trait anxiety, and trait anger. In the same vein, the accuracy ratings for the displayed basic emotions (disgust, anger, fear, sadness) did not differ between the groups. The intensity ratings for the emotion categories were also comparable with the exception of sadness. Here, the patients reported slightly higher scores, which is at odds with the assumption of an affect recognition deficit or affective blunting in PD. Our findings replicate and extend earlier reports on unimpaired facial emotion processing in PD [8–10].

In line with the subjective ratings, the brain activation in the emotion conditions only showed minor deviations between patients and controls. However, an interesting pattern emerged. The clinical group showed a generally stronger activation in somatosensory regions during emotion processing compared to the healthy participants. Several authors have argued that recognizing emotions from facial expressions requires somatosensory cortices (e.g., [26]). We decode emotional states of others by internally generating somatosensory representations that simulate how they feel when displaying a certain facial expression.

The conducted correlation analyses are in line with this assumption. In the clinical sample we observed a positive association between intensity as well as accuracy ratings for facial expressions of disgust, anger and fear and somatosensory activation. Obviously SII recruitment assisted facial emotion recognition.

The somatosensory cortex is however not the only structure relevant for affect recognition. A large number of different structures participates in this process such as the amygdala, the insula, prefrontal areas, the basal ganglia, the cerebellum, and occipito-temporal cortices [27, 28]. The basal ganglia, which show bidirectional connections with the somatosensory cortex, are one of the first targets of neurodegeneration in PD. In this context, increased somatosensory activation might function as a compensatory mechanism for reduced striatal activation. Although we did not observe a general reduction of basal ganglia activation in the clinical sample, putamen activation was lowered in the sadness condition. Here, the patients had shown the most pronounced SII involvement. In accordance with this, our patient sample particularly showed high accuracy scores for sad faces.

Our assumptions are partly congruent with findings by Anders et al. (2012) [13] who described compensatory (increased) activity in the ventrolateral premotor cortex during positive emotion processing in Parkin mutation carriers. We only investigated negative facial expressions, which are more difficult to decipher than positive ones. Consequently, this type of decoding accuracy is more frequently impaired in PD (e.g., [2, 4]). Compensatory activation of intact brain regions therefore should be more essential for improving the decoding accuracy for negative facial expressions.

Although the homogeneity of the clinical group concerning disease stage, absence of medication and comorbidity is a clear asset of our investigation, longitudinal studies are urgently needed to substantiate our findings on increased somatosensory activation in PD.

In conclusion, our study demonstrated that unimpaired emotion perception is possible in PD even with longer symptom duration and underlines the importance of controlling confounding variables which influence facial affect recognition.

## Supporting Information

S1 Table Brain activation within the control and patient group. (DOCX)
