# Supplementary material for: Facial Emotion Recognition in Parkinson's Disease: An fMRI Investigation
Source: PLoS One. 2015 Aug 18;10(8):e0136110. doi: 10.1371/journal.pone.0136110 (PMC4540566; doi:10.1371/journal.pone.0136110)
Supplement: S1 Table — (DOCX) [file pone.0136110.s001.docx]

**Table S1** Brain activation within the control and patient group

|  | H | X | Y | Z | *T* | *P*(FWE) | CS |  |
| --- | --- | --- | --- | --- | --- | --- | --- | --- |
| Controls |  |  |  |  |  |  |  |  |
| Anger > Neutral |  |  |  |  |  |  |  |  |
| MPFC | R | 6 | 42 | -15 | 3.78 | 0.020 | 46 |  |
| Pallidum | L | -21 | -9 | 3 | 3.01 | 0.048 | 26 |  |
| Fear > Neutral |  |  |  |  |  |  |  |  |
| Amygdala | L | -21 | 0 | -15 | 3.01 | 0.044 | 7 |  |
| MPFC | R | 6 | 54 | -9 | 3.33 | 0.048 | 27 |  |
| Disgust > Neutral |  |  |  |  |  |  |  |  |
| inferior frontal gyrus | L | -51 | 30 | 3 | 2.91 | 0.037 | 18 |  |
| Sadness > Neutral |  |  |  |  |  |  |  |  |
| ACC | R/L | 0 | 30 | -6 | 3.90 | 0.044 | 45 |  |
| ACC | R | 3 | 33 | -9 | 3.88 | 0.039 | 28 |  |
| Secondary somatosensory cortex | L | -45 | -15 | 24 | 4.14 | 0.007 | 43 |  |
| Patients |  |  |  |  |  |  |  | |
| Anger > Neutral |  |  |  |  |  |  |  | |
| OFC | R/L | 0 | 54 | -21 | 6.75 | 0.002 | 68 | |
| MPFC | L | -3 | 54 | -21 | 6.16 | 0.001 | 42 | |
| Inferior parietal lobule | R | 48 | -33 | 21 | 2.92 | 0.042 | 12 | |
| Inferior Frontal Gyrus | L | -51 | 33 | 6 | 3.15 | 0.030 | 19 | |
| Inferior Frontal Gyrus | R | 54 | 30 | 3 | 3.27 | 0.022 | 18 | |
| Fear > Neutral |  |  |  |  |  |  |  | |
| Inferior Frontal Gyrus | L | -51 | 30 | 0 | 3.07 | 0.030 | 15 | |
| Disgust > Neutral |  |  |  |  |  |  |  | |
| OFC | L | -9 | 57 | -18 | 3.97 | 0.037 | 62 | |
| MPFC | L | -6 | 51 | -15 | 3.60 | 0.035 | 56 | |
| Inferior parietal lobule | R | 66 | -39 | 24 | 3.82 | 0.028 | 90 | |
| Secondary somatosensory cortex | L | -48 | -21 | 21 | 3.71 | 0.037 | 49 | |
| Inferior Frontal Gyrus | L | -51 | 30 | 0 | 3.21 | 0.024 | 15 | |
| Sadness > Neutral |  |  |  |  |  |  |  | |
| Secondary somatosensory cortex | R | 54 | -15 | 12 | 3.36 | 0.059 | 22* | |

* only marginal significant

H = hemisphere, MNI coordinates (x,y,z), *P*(FWE) = corrected for family-wise error, CS = cluster size
